# An Enigmatic Case of Adult-Onset Still's Disease

**DOI:** 10.7759/cureus.60822

**Published:** 2024-05-21

**Authors:** Yash Duseja, Ayondyuti Bora, Anupam Dutta, Subhalakshmi Das, Samrat Bhattacharjee

**Affiliations:** 1 Department of Medicine, Assam Medical College and Hospital, Dibrugarh, IND; 2 Department of Internal Medicine, R.C. Aggarwal Multi-Speciality Hospital, Tinsukia, IND; 3 Department of Pathology, Assam Medical College and Hospital, Dibrugarh, IND

**Keywords:** adult-onset still’s disease, quotidian fever, yamaguchi criteria, inflammatory polyarthritis, oral methotrexate

## Abstract

An inflammatory condition known as adult-onset Still's disease (AOSD) is typified by quotidian (daily) fevers, arthritis, and a transient rash. This report details the case of a 21-year-old female patient who presented to our hospital with polyarthritis, a red rash, and a high-grade fever for more than three months. History was suggestive of AOSD, which was further proved by investigation and Yamaguchi criteria for AOSD. Steroids and methotrexate were started, to which the patient responded very well and thereafter.

## Introduction

Multi-organ involvement, ephemeral rash, arthritis, and quotidian (daily) fevers are the hallmarks of adult-onset Still's disease (AOSD), an inflammatory condition with unknown etiology [1]. Three basic patterns can be identified in the clinical history of AOSD: monophasic (or monocyclic), intermittent, and chronic. The illness course of people with monophasic AOSD usually lasts only a few weeks or months, and most patients recover fully in less than a year [2]. Around the world, AOSD is a rare ailment that affects females more than males. Around 75% of patients claim that their illness began between 16 and 35 years of age [3]. The differential diagnosis for AOSD may be extensive, and the diagnosis is still one of exclusion. Drug hypersensitivity reactions, autoimmune disorders, neoplastic conditions, and infections can resemble the clinical presentation of AOSD [4].

Non-steroidal anti-inflammatory medications (NSAIDs), aspirin, glucocorticoids, and immunomodulating medications are among the available treatment choices. The majority of individuals need steroids at some point during their AOSD; 0.5-1.0 mg/kg/day is the typical dose of prednisone, and 76% to 95% of patients respond to steroid therapy [5].

## Case presentation

**Figure 1:**
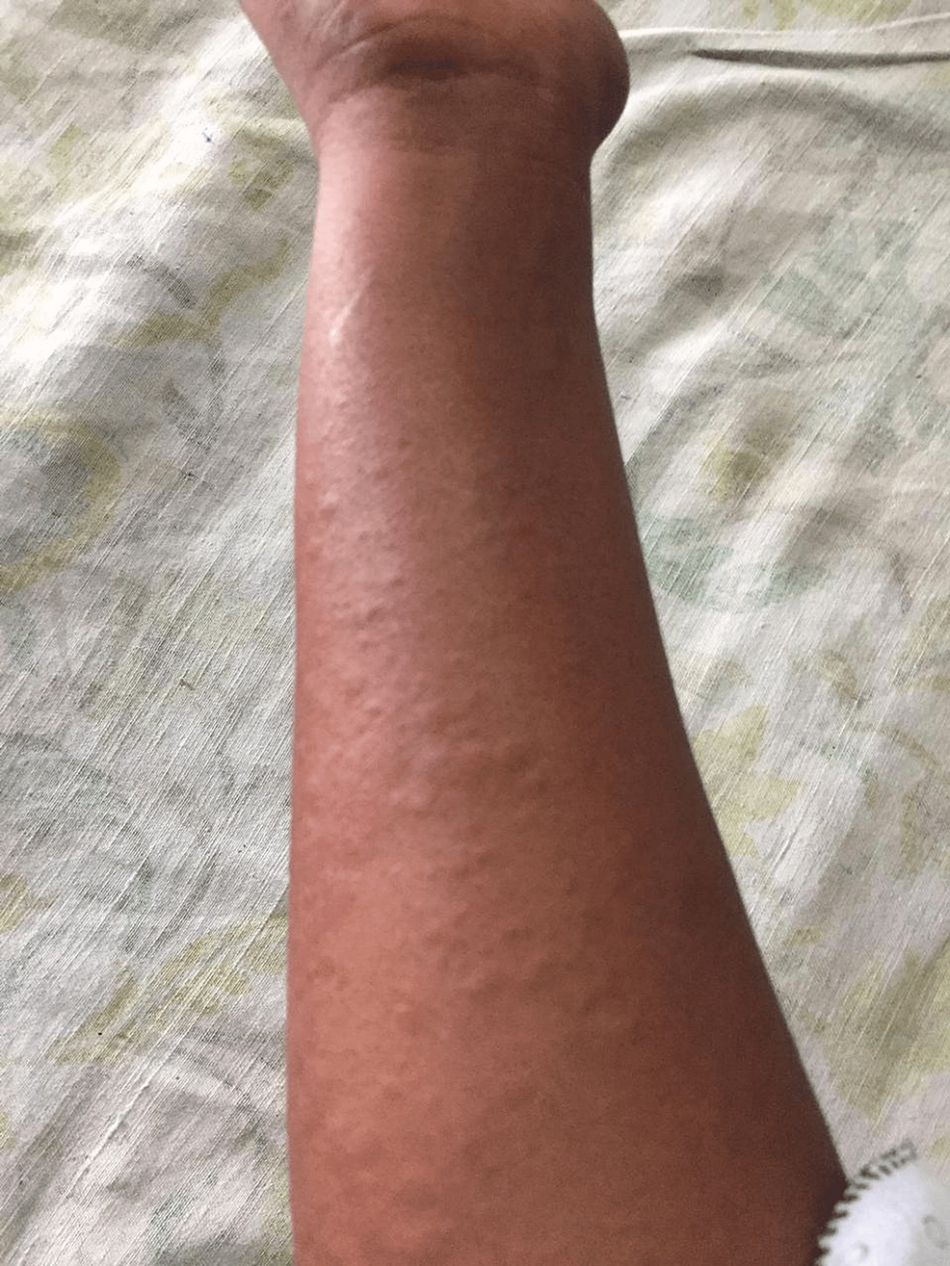
Evanescent maculopapular rash associated with Still's disease.

A 21-year-old female presented to our institute with a six-month history of fever, joint pain, and rashes that became more prominent during fever spikes. The fever was severe, peaking at 39.2°C and remaining constant throughout the day. It responded to medication and was not accompanied by any chills or rigors. The rashes were macular and maculopapular, transient, salmon-colored, and non-pruritic. They were more pronounced on her extremities and trunk (Figure 1). There was no significant history of involvement of the oral cavity or eyes or significant history of morning stiffness, prolonged cough, joint deformities, breathing difficulties, loose stools, or vomiting. There was also no prior history of similar illnesses in her family, and she had not received herbal or alternative medication.

During the clinical examination, her chest appeared normal, and the cardiovascular assessment was unremarkable. Abdominal examination revealed hepatomegaly and splenomegaly, along with enlarged lymph nodes in the inguinal region. No signs of skeletal deformity were noted. Investigations were carried out to rule out infectious or malignant causes of fever and joint pain.

Laboratory results indicated a hemoglobin level of 9.8 g/dL (reference range: 13-17 g/dL), a total leukocyte count of 25,600/cumm (reference range: 4,000-11,000/cumm), and a platelet count of 1.7 lakh/cumm (reference range: 1.5-4.1 lakh/cumm). Liver function tests showed an elevated serum glutamic-oxaloacetic transaminase (SGOT) level of 176 U/L (reference range: 14-36 U/L) and a low albumin level of 2.30 g/dL (reference range: 3.5-5.0 g/dL). The patient was evaluated for pyrexia of unknown origin, and both blood and urine cultures came back sterile. A normal 2D echocardiography ruled out any heart valve vegetation. The antinuclear antibody (ANA) profile was negative, but C-reactive protein (CRP) and erythrocyte sedimentation rate (ESR) were elevated at 79.88 mg/L (0reference range: -1.0 mg/L) and 80 mm/hour (reference range: 0-20 mm/hour), respectively. Serum ferritin levels were significantly high, exceeding 1200 ng/mL (reference range: 20-340 ng/mL). Tests for hepatitis B antigen, dengue (NS1, IgG, and IgM), malaria parasite, Typhi Dot, rK-39 antigen, leptospirosis, and scrub typhus all yielded negative results. Quantitative rheumatoid factor (RF) was low at 8 (reference range: <12.0), further excluding rheumatoid arthritis. Bone marrow aspiration and biopsy revealed myeloid hyperplasia, and BCR-ABL testing ruled out chronic myeloid leukemia.

Recurrent fever, mild rash, joint pain, myopathy, splenomegaly, hepatomegaly, lymphadenopathy, significantly low albumin with raised ESR and CRP, leukocytosis with predominant neutrophils, anemia, very high serum ferritin level, negative RF, and negative ANA test were the reasons for the suspecting diagnosis of AOSD in this patient.

She was started on prednisolone 40 mg, to which she responded well, becoming afebrile and experiencing an improvement in joint pain and skin rashes. She was discharged with a tapering dose of steroids and is currently symptom-free.

## Discussion

Still's disease, which George Still first identified in children in 1896, is the name given to systemic juvenile idiopathic arthritis [6]. The cause of AOSD is unknown; several viral triggers and genetic factors have both been proposed as significant factors, but no infectious etiology has been shown, and there has been conflicting data regarding the importance of genetic factors [5]. Human leukocyte antigen HLA-B17, HLA-B18, and HLA-B35 were linked to AOSD in a group of 62 French patients as an illustration of research on the immunogenetics of AOSD [5].

The prevalence of AOSD is 1.5 cases per 100,000-1,000,000 individuals, with equal distribution across the sexes, and the age distribution is bimodal, with two peaks: one occurs between 15 and 25 years of age, while the other occurs between 37 and 47 years of age [7]. It is only through the identification of the remarkable combinations of clinical and laboratory anomalies that AOSD may be diagnosed [8]. In the absence of bacterial or viral symptoms, this should raise suspicions of AOSD [8]. Around 70% of individuals with AOSD had significantly increased blood ferritin values [8].

The Yamaguchi criteria (Table 1) is used to diagnose AOSD along with high ferritin levels [9]. Inflammation can be lessened by non-steroidal anti-inflammatory medicines (NSAIDs), such as aspirin, ibuprofen, or naproxen [10]. AOSD patients with severe joint pain and high fever spikes have responded well to glucocorticoids, such as prednisone (0.5-1 mg/kg/day) and methotrexate in a rare number of cases [11]. Infliximab and adalimumab, belonging to tumor necrosis factor inhibitors (TNF-alpha) along with IL-1 and IL-6 inhibitors, are effective to some extent [12].

**Table 1:** Yamaguchi criteria ANA, antinuclear antibody; PMN, polymorphonuclear leucocyte; WBC, white blood cells

Major criteria	Minor criteria
Fever >39°C for >1 week	Sore throat
Arthritis/arthralgia for >2 weeks	Lymphadenopathy
Typical rash	Abnormal liver enzymes
WBC>10,000 with 80% PMN	Negative ANA

## Conclusions

In cases of pyrexia of unknown origin, especially when a patient presents with polyarthritis, a skin rash that lasts longer than two weeks, and high-grade intermittent fever, it is important to consider the possibility of an AOSD diagnosis. To rule out further AOSD differentials such as acute or chronic infections, autoimmune diseases, vasculitis, and malignant disorders, the patient should undergo a thorough evaluation.
